# Does quality of life feedback promote seeking help for undiagnosed cancer?

**DOI:** 10.1007/s11136-020-02431-7

**Published:** 2020-03-26

**Authors:** Suzanne M. Skevington, Hannah Long, Nicola Gartland

**Affiliations:** Division of Psychology and Mental Health, Faculty of Biology, Medicine and Health, International Hub for Quality of Life Research, Manchester Centre for Health Psychology, University of Manchester, Manchester, M13 9PL UK

**Keywords:** Cancer, Quality of life, Feedback, Delay, Oncology, Community, Public health

## Abstract

**Purpose:**

Diagnosing cancer early is an imperative, as help-seeking delays affect survival. Quality of life (QoL) deteriorates *after* diagnosis, but decline may start when cancer is suspected at the earliest stage of the pathway to treatment. This study examined whether offering guided feedback about personal QoL to adults with potential cancer symptoms, living in deprived communities, changes QoL and promotes help-seeking in primary care.

**Methods:**

Visitors to a CRUK mobile cancer roadshow were recruited in 43 sites. A prospective longitudinal (2 × 2) repeated-measures design was applied. Where they presented a potential cancer symptom, and were ‘signposted’ to a GP, they were allocated to a *symptom* condition, or a *lifestyle* condition, if seeking cancer risk advice. Randomisation was to an *Intervention* group, who received feedback about personal QoL results (WHOQOL-BREF and WHOQOL importance measures), or a *Control group* who assessed QoL without feedback. Depression was screened.

**Results:**

Of 107 participants, the mean age was 53; 50% were women, 57% were without tertiary education, 66% were unemployed and 45% were currently ill. Over 10 weeks, 54% of all those with symptoms sought help from a medical source and 42% specifically from a GP. Thirty-one completed all three assessments. With symptoms present, psychological, social and environmental QoL were poor, becoming poorer over time. When the symptoms group received feedback, psychological QoL increased, but GP visits were unaffected. However, feedback increased help-seeking from informal social contacts. Lifestyle groups reported consistently good psychological and social QoL.

**Conclusion:**

This early cancer research offers practical and theoretical implications for QoL interventions in deprived communities.

## Purpose

Early diagnosis improves cancer prognosis [1, 2] and is an intervention priority [3, 4]. As cancer is commonly diagnosed in primary care [5], the timing of the first consultation is crucial to reducing delays in treatment [2]. Although the pathways to treatment model [6] demonstrates transitions throughout the cancer ‘journey’, there is little information about the pre-diagnostic stage when symptoms and signs are first detected. Recognition that symptoms signal cancer could damage quality of life (QoL), and may influence the timing of consulting a General Practitioner (GP). Following cancer diagnosis, QoL deteriorates, e.g. [7], but this decline may start as soon as cancer is suspected. As community access is difficult during this period, empirical evidence is scarce. We examine whether QoL deteriorates at the earliest stage of the pathway, when bodily changes raise cancer awareness before consulting primary care.

Interventions feeding-back personal QoL results to patients and professionals can improve clinical communications, promote shared decision-making and guide self-management of health. Pioneering feedback interventions showed equivocal well-being improvements [8–10], but technique refinements have improved emotional health in cancer [11, 12] and other conditions [13, 14]. Feedback mechanisms are poorly understood [15–17], but non-clinical research [18–21] is clarifying processes previously obscured by chronic disease. For instance, community adults report modest improvements to psychological QoL after personal feedback about QoL and its importance [20–22]. Its evaluation pointed towards increased motivation to attend primary care, although good outcomes for depressed participants remained uncertain [21]. Guided by self-regulation theory [23], this process resonates with the World Health Organisation’s definition of QoL which implies that comparisons are used to judge QoL goals: ‘An individual’s perception of their position in life, in the context of the culture and value systems in which they live, and in relation to their goals, expectations, standards and concerns’ [24].

The present study aimed to investigate whether feeding-back personal QoL information could change QoL when a potential cancer symptom is present. Furthermore, whether poor QoL at the time of increasing cancer awareness promotes primary care attendance. It was predicted that QoL would decrease when certainty about cancer increased, after being ‘signposted’ to a GP; also that those with symptoms would report poorer QoL than symptom-free controls. Feedback was expected to slow, arrest or possibly improve deteriorating QoL, by actively promoting self-management of health. It was predicted that psychological QoL would change in the symptoms group who received feedback, compared with symptom controls. Changes in other domains were explored.

This research has implications for cancer survival, public health and community care in socio-economically deprived settings. Previous work suggestst that high-risk cancer groups with low socio-economic status should be identified before delivering cancer care, as they appear less able to mobilise psychological resources when facing a cancer diagnosis [25]. Rates of early-stage cancer diagnosis in deprived regions of Manchester (50%) and Salford (56%) are significantly worse than the UK average (59%) [26] https://www.cancerresearchuk.org/sites/default/files/local-cancer-stats. This provides our study context.

## Methods

### Sampling and recruitment

Adults were recruited at Cancer Research UK’s (CRUK) North West regional summer roadshow (2015/2016) in 43 socio-economically deprived urban locations across Greater Manchester. Weekly advertising publicised the roadshow mobile location in high streets and shopping centres. Specialist nurses offer services (e.g. body mass index, ‘smokerlyser’), private conversations and health messages, to increase cancer awareness. They do not diagnose, but ‘signpost’ visitors to a GP, where appropriate. The CRUK team were briefed about the research; after the visit, they offered introductions to the researcher (NG), who recruited, interviewed and assessed, in an adjacent tent.

### Procedure

The inclusion criteria were: (i) a bodily change (sign/symptom) signifying a potential cancer (e.g. non-healing sore, unexplained bleeding/cough/lump) OR (ii) seeking lifestyle advice to reduce cancer risks (e.g. alcohol, smoking, diet, sun, exercise, weight control, screening). Exclusion criteria were: (i) having already reported the current symptom/sign to a doctor/GP, (ii) a concurrent cancer diagnosis, and (iii) exclusive concern about another persons’ health. Potential participants were screened for age (> 18 years), fluent English, no visual difficulties (eyewear permitted) and depressive symptoms.

### Study design and development

A prospective randomised controlled design with repeated measures was applied. The initial 2 × 3 design contained six cells: two conditions and three intervention groups. The conditions were a *symptom group,* where the symptom/sign was a potential cancer, and a *lifestyle* group seeking advice to avoid cancer. A block randomisation list allocated participants to the *intervention* group or one of two *control* groups (ratio 1:1:1) (https://www.sealedenvelope.com/simple-randomiser/v1/lists). The full, guided feedback package received by the *intervention* group included inspecting QoL results with guided interpretation, planned self-management and resource information. Group 1 *controls* did not receive QoL questionnaires or feedback. Group 2a controls just completed the measures without feedback etc. (see Table 1). Evaluations were conducted at baseline, then two and 10 weeks after baseline.

**Table 1:** Total sample breakdown, intervention groups and conditions, with age and gender

	Conditions		
	Lifestyle	Symptoms	Total
**Baseline (time 1) at 0 weeks**			
(1) No intervention	24 (*F* = 11, < 45 = 2)	11 (*F* = 4, < 45 = 5)	35
(2a) QoL measurement#	8 (*F* = 5, < 45 = 1)	4 (*F* = 2, < 45 = 1)	12
(2b) Reallocated*	7 (*F* = 4, < 45 = 2)	14 (*F* = 6, < 45 = 3)	21
(3) QoL feedback	22 (*F* = 11, < 45 = 4)	17 (*F* = 10, < 45 = 2)	39
Baseline totals	61	46	107
**Follow up 1 (time 2) at 2 weeks**			
QoL measurement only controls#	24 (*F* = 10, < 45 = 2)	15 (*F* = 7, < 45 = 7)	39
QoL feedback intervention	16 (*F* = 9, < 45 = 3)	13 (*F* = 6, < 45 = 0)	29
Follow-up 1 totals	40	28	68
**Follow up 2 (time 3) at 10 weeks**			
QoL measurement only controls	22 (*F* = 10, < 45 = 1)	9 (*F* = 4, < 45 = 3)	31
QoL feedback intervention	12 (*F* = 7, < 45 = 1)	11 (*F* = 3, < 45 = 0)	23
Follow-up 2 totals	34	20	54
**Testing intervention over time**			
QoL measurement controls	5	6	11
QoL feedback intervention	12	8	20
Totals	17	14	31

At both Follow-ups, one single Control group (#) was formed by combining groups 1 and 2b with 2a, for comparison with the QoL feedback Intervention group—see “Methods”

*F* female; < 45 = younger than 45 years

*Reallocated to 2a due to higher depression

This piloted, modified design was adjusted. Due to low recruitment, control group 1 was discontinued during baseline (Table 1). As no statistical differences were found between controls 1 and 2a on key variables, and neither group received feedback, they were merged, and randomisation adjusted. Intervention participants reporting moderate/severe depression at baseline (Group 2b) (PHQ-2 scores > 3) were reallocated to Group 2a, due to ethical concerns that feedback might increase depression; lost numbers were replaced. Persistent low recruitment resulted in further randomisation adjustments (1:1.5) to increase intervention numbers. Thereafter, one single control group was formed by combining all participants whose QoL was assessed without feedback at baseline, i.e. groups 1, 2a and 2b (see Table 1). The final 2 × 2 design therefore contained four groups: one intervention (feedback), one single, combined control, and two conditions: symptom and lifestyle. Results are reported for symptom intervention, symptom control, lifestyle intervention and lifestyle control cells.

### Guided QoL feedback intervention

**Fig. 1:**
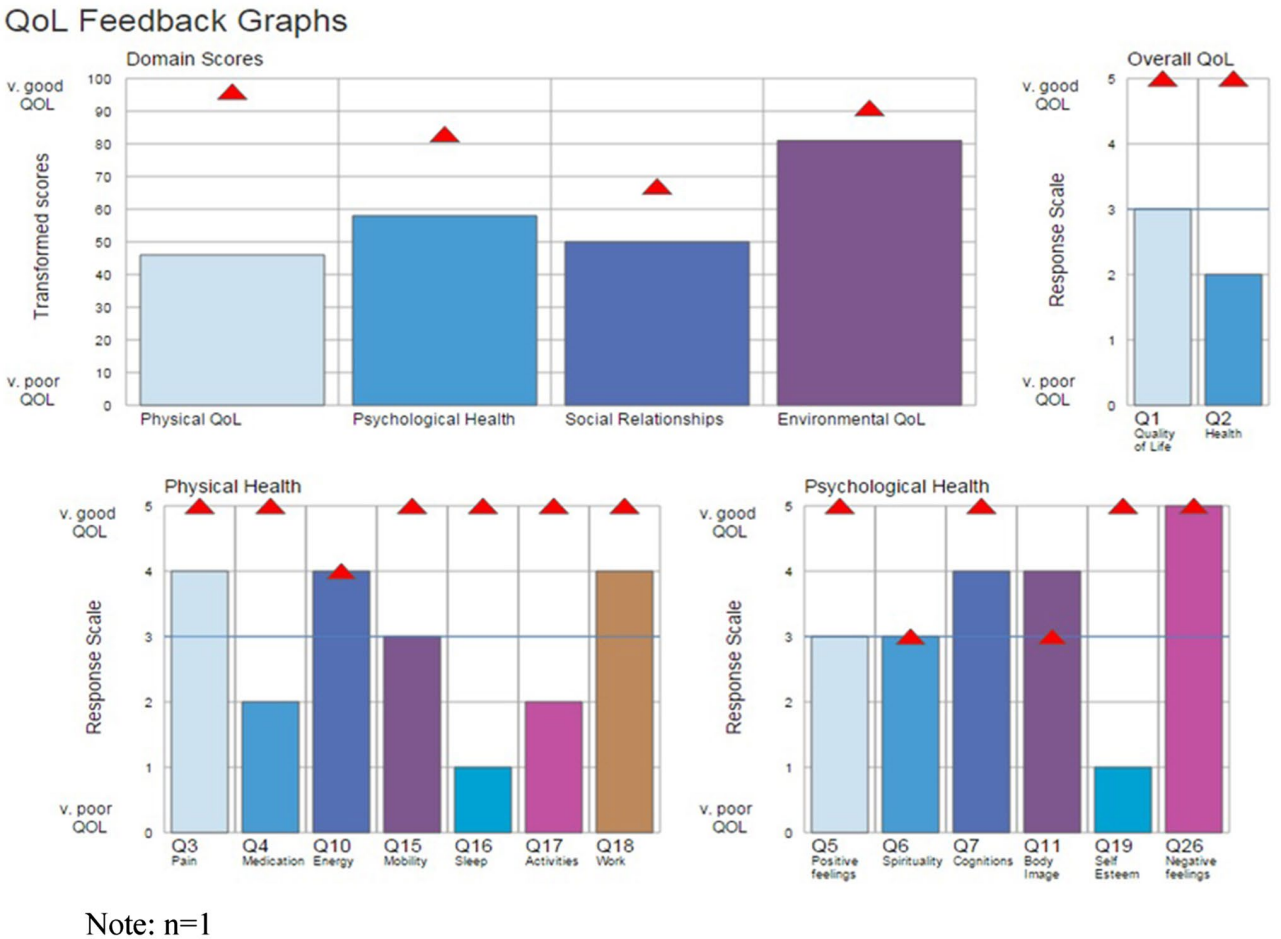
An extract of the UK WHOQOL-BREF feedback graphs illustrating facet profiles of physical and psychological domains only, domain scores, general QoL and facet importance ratings. Red triangles indicate the importance of QoL. (Color figure online)

To improve self-management, individualised feedback of personal QoL results was presented to the intervention group using a computerised graphical summary [21]. WHOQOL-BREF profiles of QoL facets (dimensions) and their importance ratings were inspected simultaneously. In Fig. 1, a red triangle representing importance is superimposed on a coloured bar indicating facet QoL level; high scores mean good QoL. A semi-structured schedule was followed throughout feedback and interpretation. First, scores for domains (0–100 scale) and general QoL and health (1–5) were explained. Scores above 50 (okay) reflect good/very good QoL and < 50 reflect poor/very poor QoL. Next facets showing good QoL were inspected: high ratings (4/5) indicated good QoL and low ratings (2/1) poor QoL. Then, facets with high importance (4/5) and low importance (2/1) were considered. Where a particular facet showed *both* poor QoL *and* high importance [21, 22], a large gap (two or more points) was visible, as illustrated by self-esteem (Q19), sleep (Q16), activities of daily living (Q17) and dependence on medication/treatments (Q4), as shown in Fig. 1. For every pinpointed facet we inquired the following: (i) How could this aspect of QoL be improved? (ii) What resources would be needed to make changes? (iii) What practical action(s) are needed to address *poor and important* aspects of QoL? Inspecting facets with high QoL and high importance provided a positive conclusion. Finally, a list of resources was offered, e.g. Age-UK, MIND, drugs/alcohol, bereavement and Citizen’s Advice.

All procedures accorded with ethical standards of Univ. of Manchester (Faculty of Medicine; Ref. 15,163) and the 1964 Helsinki Declaration with its later ethical standards. Fully informed, written consent and contact information was obtained from all participants. Consent was reaffirmed at each follow-up when the unique baseline identification code was regenerated. Baseline socio-demographic information included birth date, gender, marital status, ethnicity, highest educational level, employment status and self-reported health. The participant or researcher (NG), entered replies into a laptop, on-site; data were transmitted to a secure server. Follow-ups were conducted via email, post or phone, as preferred, using a bespoke website or paper. Non-response after 3 weeks resulted in two further weekly approaches. Follow-up questionnaires were sent to all baseline recruits; they were identical for all intervention groups, except for the addition of body change and symptom-related help-seeking questions in the symptoms pack. The intervention and roadshow were separately evaluated.

### Materials

(i) *The World Health Organisation’s Quality of Life Assessment (WHOQOL-BREF)* assess subjective QoL applied to health [24] with 26 items. Two general items rate overall QoL and health; the other 24 are scored in a physical, psychological, social or environmental QoL domain. Five-point Likert, interval rating scales assess QoL over the past two weeks. Raw domain scores (range 4–20) are transformed onto a 0–100 scale. The WHOQOL-BREF shows good international reliability and validity [27, 28], including in UK [27, 29]. Scores respond sensitively to clinical and social change [30, 31].
(ii) *WHOQOL Importance* International items assess the importance of 25 WHOQOL-BREF facets on 5-point Likert importance scale (e.g. *How important is it to you to be free from pain?*) without a time frame. It shows good internal consistency, construct validity and stability [22, 32–34]. A shortened form was used [22].
(iii) *Patient Health Questionnaire-2 (PHQ-2)* As depression reduces QoL [30, 35], we used the PHQ-2 to screen for depressed mood/anhedonia, over the two weeks before baseline [36]. Two interval ratings provide score totals from 0–6; > 3 indicates depressive symptoms present. A depression covariate was analysed.
(iv) *Interview.* Bodily changes were qualitatively coded from CRUK’s cancer symptoms/signs list (https://www.cancerresearchuk.org/about-cancer/cancer-symptoms).
(v) *Help-seeking* We asked about seeking *formal* help from a GP for a symptom/sign, between baseline and follow-ups. *Informal* sources of help, and concurrent health concerns, were noted. All sources of help were coded: (i) *health*, e.g. doctor/GP, pharmacist, cancer screening, emergency services; (ii) *social*, e.g. intimate partner, friend, family, clergy; (iii) *remote*, e.g. Internet, help-line, charity (e.g. CRUK).
(vi) *Evaluations* A 5-point rating scale evaluated roadshow satisfaction two weeks after baseline (FU1): ‘How satisfied were you with the service provided by the roadshow*?*’: ‘not at all satisfied’ (1) to ‘very satisfied’ (5) [37]. At 10 weeks (FU2), the intervention was evaluated: *‘*How useful was participating in the ‘Quality of life and Health of the Community research’ project, for you?’: ‘not at all useful’ (1) to ‘very useful’ (5), followed by the open-ended question, ‘In what ways?’

### Analysis

Raw domain scores from WHOQOL measures were transformed, and a general QoL mean was calculated (SPSS v.22). Baseline follow-up differences were calculated; other baseline results are reported elsewhere [38]. Testing interval variables for normality most showed acceptable skew and kurtosis. Minor positive skew was found for social facets (baseline); social support and transport (FU1); information, mobility, home environment, health/social services and pain (FU2). Kurtosis for finance and leisure (FU1) was noted. Log transformations marginally improved normality, so original scores were analysed. Sample means were imputed. Correlations between the dependent and independent variables also investigated potential covariates. *χ*^2^ and *t* tests of socio-demographic variables compared intervention group differences by domains, then conditions, and general QoL. When evaluating intervention impact, intervention and control groups were compared, then symptom vs lifestyle conditions, and repeated for roadshow satisfaction.

To further assess QoL group differences (2 × 2), multivariate analysis of co-variance (MANCOVA) with repeated measures over time was conducted by domain. Intervention groups were split into lifestyle and symptom conditions, with depression as the covariate. Significance was expected for intervention x condition interactions, and intervention main effects (*p* < 0.05). Estimated marginal means (EMMs) adjusted for depression. Box’s *M* tested the homogeneity of DV variance (rejection *p* < 0.001) and Mauchly’s *W* sphericity (rejection *p* < 0.05) [39].

To assess the intervention impact on help-seeking, *χ*^2^ compared frequencies of seeking, and not seeking help in symptom feedback, and no feedback controls. More formal GP help-seeking was expected for the symptom feedback, but not lifestyle group. Independent t tests compared help-seeking from informal sources by domain, in symptom and lifestyle conditions.

## Results

### Sample description

Of 215 roadshow visitors, 131 were approached and 109 enrolled. Two withdrew during completion, so the baseline total was 107. Time constraints, commitments to others and lacking interest covered main refusal reasons. After two weeks, 68/107 completed. After 10 weeks, 54/107 completed (missing lifestyle data 3, symptoms 1). Missing facet data were 0.4% at FU1 and 1.2% at FU2. No help-seeking information was reported by 8 lifestyle and 2 symptom participants—FU1, and 3 lifestyle and 1 symptoms participant—FU2. A subset of *N* = 31 provided full information on all three occasions; these are analysed to test the intervention (see Table 1). As Group 1 (*N* = 35) did not complete any QoL measures at baseline, they are not in the analysis.

Of 107, 50% of each gender were recruited at baseline; 49% men were retained at FU2. Mean age was 53 years; 96% were ethnically white. Primary or secondary education alone was completed by 57%—high for UK (51%) [40]. Unemployment at 66% was exceptionally high compared with the Greater Manchester region (7.3%), and UK (6%) [41]. Most were single (42%) or married (39%). Forty-five % were currently ill and 96% had one or more chronic illnesses. Twenty-six % reported high depressive symptoms (> 3.0) at baseline, but 11% at FU2. The symptom condition had higher depression (45%) than lifestyle (24%). Controls had higher depression than the intervention (*t*_(54.3)_ = 3.07, *p* < 0.01), due to reallocation. Depression remained high at FU1 (*t*_(38.9)_ = 2.09, *p* < 0.05), but not FU2 (*p* > 0.05). At FU2, fewer of the intervention group were employed (*χ*^2^_(1)_ = 5.69, *p *< 0.05). Due to small intervention cell numbers, and to maximise analysing all collected data, some analyses as appropriate are conducted on the whole group. Baseline symptoms were classified as follows: unexplained ache/pain (24%); unhealed sore (15%); unusual lumps, breast change (11%); changing mole, bowel habit (9%); faecal blood, persistent cough (9%); urinary (6.5%); and unexplained weight change (6.5%). However, not everyone who reported a bodily change suspected cancer, e.g. when seeking obesity advice, one incidentally reported bowel changes.

### Evaluation

The ‘satisfaction with the roadshow’ evaluation was completed by 49/68 participants who also completed all other FU1 measures. These and the research intervention ratings (FU2) (*N* = 40/54) were compared for interventions and conditions. All ANOVAs were non-significant (*p* > 0.05), indicating that similar satisfaction levels did not differentially affect other results.

After evaluating the feedback intervention, 17 provided comments. Four key themes emerge from content analysis (Table 2): (i) Confirmation that the intervention was successful in promoting health and well-being, with subthemes on: direct effects of feedback, motivation to be healthy and time for personal reflection; (ii) Additional new benefits to individuals: resilience to adversity, assistance in achieving other goals and awareness of choices; (iii) Benefits to the community and social groups: community value, gratitude and attention to the environment; and (iv) Altruistic reasons for participation. Evaluation orientations ranged from neutral to positive.

**Table 2:** Evaluation of the intervention from participants who received feedback

**Confirmation that the intervention promotes health and well-being**	
Promotes primary care attendance: ‘Made me visit the doctor’	
Direct effect of feedback: ‘I have noticed that I feel slightly better about my health after talking to [nurse] and [researcher]’	
Motivated to be healthy: ‘I try to lead an active life and eat sensibly’	
Provided time for personal reflection: (i) ‘Made me stop and think of my personal well-being and environment’ (ii) ‘Gave me a chance to take note of myself.’ (iii) ‘Good in some ways, it’s helping me. It’s sort of like talking to someone and listening and helping’	
**Additional new benefits of the intervention to individuals**	
Resilience: ‘Made me realise that a positive attitude is of great benefit when dealing with adversity’	
Helps achieve other personal goals: ‘Helping I make sure I look after myself so I can see my children’	
Aware of choices: ‘Made me appreciate all the more my health, and the ability to choose how I live my life’	
Distraction: ‘It kept me busy’	
**Benefits to community and social groups**	
Community value: ‘To learn that both these issues (health and QoL) are very important in our community, and with peoples’ help, they can be made better’	
Gratitude: ‘It made me aware of how lucky I am to have good health, and a lovely family life’	
Attending to the environment: ‘It made me think about my circumstances and how I feel about myself’	
**Altruistic reasons for participation**	
Helping others/researcher: (i) ‘I like to think I am helping people’ (ii) ‘I am hoping it will help you’	

### Examining QoL differences between interventions and conditions for each domain

Interventions and conditions were compared by domain, for the 31 participants in Table 1, who completed on all three occasions (MANCOVA; Table 3). Overall, psychological QoL was significant (Pillai *F* = 3.83 (df 2) *p* = 0.035, *η* 0.24) and acceptable (Box’s *M*, *p* = 0.066); Mauchly’s *W* required Greenhouse–Geisser correction (*p* = 0.74).

**Table 3:** Quality of life in four WHOQOL-BREF domains for the feedback intervention vs no feedback controls and symptom vs lifestyle conditions (*N* = 31)

Intervention groups/time	WHOQOL domains					
	Physical QoL			Social QoL		
Baseline	Lifestyle	Symptoms	Total sample	Lifestyle	Symptoms	Total sample
Control	54.29 (28.9)	36.90 (19.6)	44.80 (24.7)	66.67 (33.3)	48.61 (24.4)	56.81 (28.8)
Intervention	76.74 (14.6)	56.25 (19.2)	68.54 (19.1)	70.14 (23.4)	54.17 (23.6)	63.75 (24.2)
Total	70.13 (21.6)	47.95 (21.1)	60.12 (23.8)	69.12 (25.6)	51.79 (23.2)	61.29 (25.7)
**Follow-up 1 at 2 weeks**						
Control	49.40 (30.3)	33.82 (13.4)	40.90 (22.9)	60.00 (29.1)	29.86 (23.0)	43.56 (29.2)
Intervention	75.35 (16.9)	50.89 (21.3)	65.56 (22.0)	71.87 (18.2)	39.58 (20.3)	58.96 (24.6)
Total	67.71 (23.9)	43.58 (19.8)	56.81 (25.0)	68.38 (21.7)	35.41 (20.8)	53.49 (26.9)
**Follow-up 2 at 10 weeks**						
Control	52.86 (25.4)	31.55 (20.4)	41.23 (24.3)	65.00 (27.3)	27.77 (11.4)	44.69 (27.2)
Intervention	76.98 (13.4)	48.95 (25.8)	65.77 (23.4)	66.67 (17.8)	35.41 (20.8)	54.17 (24.3)
Total	69.88 (20.4)	41.45 (24.5)	57.07 (26.2)	66.18 (20.1)	32.14 (17.3)	50.80 (25.0)

Intervention groups/time	WHOQOL domains					
	Psychological QoL			Environmental QoL		
Baseline	Lifestyle	Symptoms	Total sample	Lifestyle	Symptoms	Total sample
Control	55.00 (17.0)	41.66 (35.1)	47.72 (27.9)	61.25 (11.2)	57.29 (17.4)	59.09 (14.4)
Intervention	76.04 (11.1)	53.13 (13.3)	66.87 (16.4)	78.38 (18.2)	63.28 (25.4)	72.34 (20.5)
Total	69.85 (16.0)	48.21 (24.6)	60.08 (22.7)	73.35 (17.9)	60.71 (21.8)	67.64 (17.1)
**Follow-up 1 at 2 weeks**						
Control	50.83 (18.9)	36.80 (17.6)	43.18 (18.8)	49.38 (11.8)	44.79 (21.4)	46.88 (17.1)
Intervention	75.69 (13.3)	41.15 (15.5)	61.87 (22.2)	75.52 (16.9)	65.23 (16.6)	71.41 (17.2)
Total	68.38 (18.6)	39.29 (16.0)	55.24 (22.6)	67.83 (19.6)	56.47 (20.9)	62.70 (20.6)
**Follow-up 2 at 10 week**s						
Control	50.00 (13.8)	30.28 (19.9)	39.24 (19.5)	56.25 (14.9)	47.91 (11.1)	51.71 (13.1)
Intervention	79.38 (13.9)	40.13 (14.3)	63.75 (23.9)	76.04 (15.7)	57.36 (26.1)	68.57 (21.9)
Total	70.74 (19.3)	36.01 (17.0)	55.05 (25.1)	70.22 (17.6)	53.32 (20.9)	62.59 (20.7)

*MANCOVA* means (standard deviation) repeated measures, and a covariate of depressive symptoms (PHQ-2)

Transformed domain scores ranged from 0 to 100; high scores mean good QoL

A group difference in psychological QoL between the intervention and controls was not confirmed as significant. Psychological QoL was poorer for the symptom than lifestyle condition, as predicted (*F* = 4.79, df 2, *p* = 0.017, *η* = 0.28). Furthermore, a significant intervention × condition interaction for psychological QoL was confirmed, as the symptom intervention (feedback) group reported better QoL than symptom controls (*F* = 6.35, df 1, *p* = 0.018, *η* = 0.19), but not for lifestyle. The depression covariate was significant (*F* = 18.78, df 1, *p* = 0.0001, *η* = 0.42). With symptoms present, poor QoL (< 50) became poorer over 10 weeks, but in the lifestyle condition remained very good (Pillai: *F* = 5.20, df 2, *p* = 0.013, *η* = 0.29). This reduction was greater at FU2 (*p* = 0.009) than FU1 (*p* = 0.047).

Overall social QoL was significant and acceptable (Pillai *F* = 4.96, df 2, *p* = 0.015; Box’s *M*, *p* = 0.47; Mauchly’s *W*, *p* = 0.96). Although intervention group differences were non-significant, better social QoL was found for lifestyle than symptoms (*F* = 4.56, df 2, *p* = 0.042, *η* = 0.149), but the interaction was non-significant. Social QoL declined over time at both follow-ups (Pillai *F* = 7.56, df 2, *p* = 0.003; FU1, *p* = 0.028; FU2, *p* = 0.002).

Although environmental QoL was significant overall (Pillai *F* = 4.69, df 2, *p* = 0.019, *η* = 0.27), no group differences were found, but environmental QoL declined at FU1 (*p* = 0.035) and FU2 (*p* = 0.04). All physical domain and general QoL results were non-significant. After disaggregating two general QoL items, an exploratory test showed significant change in subjective health only (Pillai *F* = 4.86, *p* = 0.016, *η* = 0.28), and improvements over 2 weeks (FU1, *p* = 0.025).

### Help-seeking for a potential cancer symptom

After dichotomising the symptom group into those who sought help after baseline from non-seekers, we found 15/28 with symptoms (54%) had sought help from a medical source by FU1, and 10/15 (38% of total FU1), specifically from a GP. At 10 weeks, 11/20 of the symptoms group (55%) sought medical help, and 10/11 of these (50% of total FU2) were from a GP. Over the study, 54% (26/48) of those with symptoms sought help from a medical source and 42% (20/48) from a GP.

### Does QoL differ between those who do and do not seek help within symptom and lifestyle groups?

When QoL differences were tested between conditions for those who did, or did not seek help within each condition, at follow-ups, all results were non-significant for each QoL dimension in Table 4. Help-seeking behaviour was not influenced by QoL level as predicted, although samples were small. Self-reported help-seeking behaviour and QoL was assessed in this longitudinal study, in contrast to previous cross-sectional analysis of baseline data [38] which showed that high help-seeking intentions at this time were associated with poor physical QoL.

**Table 4:** Comparing the quality of life domains of participants who did, and did not seek help from primary care, in the lifestyle and symptom conditions, 2 and 10 weeks^#^ after baseline (Independent *t* tests)

WHOQOL domain	Two weeks after baseline (follow-up 1)			
	Sought help (*N* = 5) M (SD)	No help (*N* = 8) M (SD)	*t*(df = 11)	*p*
**Lifestyle group** (*N* = 12)				
General QoL	3.80 (.76)	4.06 (.90)	.54	.600
Physical	74.29 (14.59)	78.65 (18.01)	.45	.659
Psychological	74.17 (16.24)	80.73 (74.17)	.86	.406
Social	65.00 (23.12)	79.69 (15.01)	1.40	.189
Environment	73.75 (21.72)	77.73 (17.63)	.36	.723

WHOQOL domain	Two weeks after baseline (follow-up 1)			
	Sought help (*N* = 7) M (SD)	No help (*N* = 6) M (SD)	*t*(df = 11)	*p*
**Symptom group **(*N* = 13)				
General QoL	3.14 (.69)	3.08 (1.20)	− .11	.913
Physical	51.02 (23.30)	40.38 (17.43)	−.92	.378
Psychological	38.69 (21.21)	45.14 (17.16)	.60	.564
Social	38.10 (29.21)	37.50 (25.69)	−.04	.970
Environment	64.73 (21.17)	56.77 (22.57)	−.66	.525

WHOQOL domain	Ten weeks after baseline (Follow-up 2)			
	Sought help (*N* = 7) M (SD)	No help (*N* = 4) M (SD)	*t*(df = 9)	*p*
**Symptom**^**#**^**group** (*N* = 11)				
General QoL	2.64 (.99)	3.25 (.96)	.99	.348
Physical	37.24 (20.50)	58.63 (30.08)	1.42	.191
Psychological	41.43 (17.09)	37.92 (16.19)	−.33	.746
Social	40.48 (22.27)	29.17 (10.76)	−.94	.372
Environment	49.11 (20.63)	70.98 (20.96)	.13	.127

High scores mean good QoL. Transformed domain scores range from 0 to 100; General QoL ratings 1–5

^#^No data collected from Lifestyle group at 10 weeks, as help from primary care was inappropriate

Seeking GP help was also examined for symptom groups that did, and did not receive feedback, and no significant association was detected (FU1 *χ*^2^_(2)_ = 1.10, *p* = 0.578; FU2 *χ*^2^_(2)_ = 1.59, *p* = 0.452); or for lifestyle (FU1 *χ*^*2*^_(2)_ = 0.11, *p* = 9.18; FU2 *χ*^*2*^_(2)_ = 4.91, *p* = 0.086). This indicates similar help-seeking by groups over time.

### When symptoms are present, does feedback affect help-seeking in primary care, and elsewhere?

Intervention impact on informal help-seeking from three sources during follow-up was examined by condition. Help-seeking from all health sources was not different for symptom intervention and symptom controls (FU1 *t*_(14)_ = *− *2.50*, p* = 0.806; FU2 *t*_(11)_ = 0.171, *p* = 0.867). Although lifestyle groups reported no short-term difference in seeking help from all health sources, a significant longer-term difference was found (FU1 *t*_(25)_ = 0.142*, p* = 0.89; FU2 *t*_(23)_ = * − *3.80*, p* = 0.001). Means suggest more health help-seeking with feedback (1.90) than without (1.27). For informal social sources, symptom intervention and control differences showed more help-seeking after feedback (FU1 (*t*_(14)_ = * − *2.96*, p* = 0.010; FU2 (*t*_(11)_ = * − *2.76*, p* = 0.019). This specific finding supports our general prediction that the symptoms intervention group would report most help-seeking. Help from remote sources was not different for groups, or over time.

## Discussion

Although Velikova et al. [11] and others, have found that feedback can enhance well-being in diagnosed cancer patients, the present study offers new evidence that at the earliest *pre-diagnostic* stage of the cancer treatment pathway, QoL feedback  may stem QoL deterioration in those with potential cancer symptoms *before* they consult primary care. After feedback, 54% of all those with symptoms sought help from a medical source, and 42% in this subgroup specifically sought a GP’s opinion. Alternatively, from 26 instances of help-seeking during the study, 20 of these (77%) were from a GP. Being signposted to primary care conveys an unpleasant message about future morbidity and mortality. This is illustrated by the findings that psychological, social and environmental QoL declined to very low levels, as those with symptoms digested roadshow news that they might have cancer. However, increased skew  over time in the environment domain is inconsistent with this interpretation.

Following  community research [20], we expected to find that feeding-back personal QoL information with a tailored self-management plan might slow, arrest or even improve declining QoL. We confirmed that those with symptoms who received feedback reported better psychological QoL than symptom controls. Furthermore, for those with symptoms, deteriorating QoL slowed after the intervention, showing that poor QoL became less poor compared with symptom controls. But predicted interactions were only significant for psychological QoL, although we recorded similar mean trends in other domains. Replication with larger numbers is needed. After discovering that their symptom should be discussed with a GP, guided feedback appeared to offer a mild buffer against further deterioration in psychological QoL. As the intervention did not facilitate substantial QoL improvements in the symptom group, the evidence offers weak justification for routinely providing feedback to all visitors with symptoms, except as a means of stemming further deterioration. This is the first time that the impact of a guided feedback package has been investigated at this early stage of the cancer treatment pathway, and in deprived settings.

Psychological QoL is influenced by depression [30, 35], and the findings reaffirmed this. More participants than anticipated reported elevated depression, reflecting poor mental health in these communities. Of those with symptoms who were randomised to the intervention group, 20% required re-assignment to a control, due to elevated scores. Despite adjustments via screening, reallocation and covariate control, depression influenced psychological QoL findings. How this interferes with outcomes arising from feedback should be the subject of further research. Sample characteristics such as high unemployment, little education and chronic illness represent other important disadvantages that may affect the active self-management of poor QoL [25], and motivation to attend primary care [42]. However, our results were barely affected by distress from knowing a diagnosis of cancer, as only one symptom participant  received a confirmed diagnosis by study completion. Instead, our findings illustrate the considerable damage to QoL that the threat of cancer incurs, especially with depression present. This has important clinical implications for practice, and shows that a replication with non-depressed adults is also desirable.

Recruited from the same communities as those with symptoms, lifestyle participants reported better social and psychological QoL, and their good QoL was maintained throughout. Improved psychological QoL after feedback indicates minor mental health benefits to those seeking cancer risk advice. Retained numbers and satisfaction ratings suggested that feedback was relatively acceptable to the lifestyle group. New insights were gained from exploring informal sources of help, as following feedback, the lifestyle group consulted more informal health sources than those with symptoms. Such behaviour suggests that the lifestyle group continued to process ways to improve their health afterwards. Together, these findings indicate that feedback can maintain lifestyle well-being in the community. Furthermore, consulting informal sources of help was more appropriate behaviour for this healthy subgroup than medical attention. Roadshow visitors seeking lifestyle advice could be assisted by feedback if this procedure was routinely incorporated into the roadshow service.

Specific evidence of social help-seeking confirmed our general prediction that the symptoms feedback group would seek more community help. This finding raises questions about whether informal social help supplements, replaces or delays seeking formal health care. This mechanism deserves investigation to understand whether community adults are informally deterred by their social contacts from seeking GP help in these circumstances. Nevertheless help-seeking from accessible, trusted others represents an active strategy for processing unpleasant news, so contrasting with the popular passive image. While evidence shows that community help is sought by diagnosed cancer patients [43], these new findings indicate that this behaviour is already established well before diagnosis [6]. Despite intense local pressure on health services in socio-economically deprived areas of UK, and exploding Internet use globally, remote sources were consulted at similar rates by different groups. Where informal sources are accessible, they can supplement the formal support provided by primary care [20, 21]. Where informal help-seeking is appropriate, health services can target those with the greatest need, in situations where poorer population health increases demand.

Following feedback, very good QoL persisted in the lifestyle group, illustrating the value of this intervention to community adults with better mental and physical health. The WHOQOL software offers a user-friendly tool that collects and scores replies, presents attractive results, and supports guidance, with the potential to change lifestyles that could mitigate against cancer mortality long term [44]. Written instructions support reliable, independent use, without professional involvement [20, 21]. Self-completion reduces financial costs, time and staff burden, although visitors with poor literacy/low education may need a*d hoc* assistance.

Limitations include recruitment difficulties and attrition [45] that contributed to the numbers shortfall. Where sample sizes are small, findings should be regarded as indicative. Direct researcher recruitment rather than via the roadshow might have improved numbers, but was not acceptable to CRUK. After being signposted to a GP, some were distressed, knowing that cancer was increasingly probable. The timing of recruitment could therefore have inhibited participation. Elevated depression increased reluctance to complete follow-up. Without more evidence, screening for moderate/severe depression should exclude symptom participants from feedback, as depression may intensify. Despite piloting, optimal intervention timing may not have been achieved, but did confirm that examining the beginning of the pathway was feasible. For privacy, most interviews were conducted in a tent adjacent to the roadshow, where recruitment was interrupted by bad weather. These factors affected attrition; however, follow-up was administered remotely. Roadshow logistics changed after one year, interfering with transporting research equipment to sites reliably.

Recruitment was adversely affected by the complexities of living in poverty. Some deprived areas have a history of reluctance to meet health professionals. This was exemplified by up to three years delay in reporting a symptom, and even then, to the roadshow, not a GP. As the sample was predominantly white, the findings cannot be generalised to other ethnic groups. Despite many ethnic residents in this region, very few visited the roadshow, so non-participation should be carefully researched. Despite these limitations, this novel, peripatetic roadshow service designed for four UK-deprived regions offers valuable informal, appointment-free, walk-in consultations that enables passers-by, especially men, to make spontaneous decisions to attend without prior planning.

## Conclusion

This study evaluated a QoL feedback intervention through opportunistic access to ‘invisible’, deprived communities. The findings have implications for research and practice in public health, community medicine and primary care, as they offer rare theoretical and practical insights into the earliest, pre-diagnostic stage of the pathway to treatment model [6]. Despite its modest scope, this work is strengthened by a heterogeneous sample recruited in 43 sites. As many socio-economically deprived regions exhibit high cancer rates, the policy implications are also national and international. This first independent quantitative evaluation of the CRUK roadshow offers some positive findings to support the implementation of a national service roll-out. As early diagnosis in primary care affects treatment success and ultimate survival, more preventative public health interventions are urgently needed.
